# AllPlay Dance: Two Pilot Dance Projects for Children With Disability, Developed and Assessed With a Dance Studies Approach

**DOI:** 10.3389/fpsyg.2021.567055

**Published:** 2021-10-22

**Authors:** Olivia Millard, Ebony Lindor, Nicole Papadopoulos, Carmel Sivaratnam, Jane McGillivray, Nicole Rinehart

**Affiliations:** ^1^Faculty of Arts and Education, School of Communication and Creative Arts, Deakin University, Geelong, VIC, Australia; ^2^Faculty of Health, Deakin Child Study Centre, School of Psychology, Deakin University, Geelong, VIC, Australia; ^3^Faculty of Education, School of Educational Psychology and Counselling, Monash University, Clayton, VIC, Australia

**Keywords:** dance, disability, inclusion, participation, social, creative

## Abstract

AllPlay Dance is founded on a collaborative approach to research between the School of Psychology and the School of Communication of Creative Arts, both of Deakin University. The research is also undertaken in partnership with professional ballet company, Queensland Ballet. This paper describes the development and execution of two pilot projects for children with disability, utilizing a dance studies methodology. The projects were conducted in 2018 and 2019 for children with cerebral palsy (CP) and autism spectrum disorder, as part of the AllPlay Dance program. Participants with disabilities ranged in age from 7 to 12 years. As well as describing the approach to the program development, we discuss the involvement of older and more experienced buddies who were included as a method to support the participation in dance of children with disabilities. We will also describe the diffusion of authorship in the making of group dances as a tool for inclusion and the premise of dance as a social practice in which participants inter-subjectively generate meaning and sense making. The AllPlay Dance projects were developed as a series of dance classes in which participants worked with set or learned movement material, dance improvisation, and tasks for movement generation in order to collectively generate a dance for performance. This paper focuses on the aim of developing inclusive approaches to dance classes that challenge “ableist” notions of dance as spectacle to enable to work toward building transferable programs to allow all children who so desire and to participate in dance in their communities.

## Introduction

This paper discusses the development of two pilot dance projects for children with disabilities initiated as part of the AllPlay program of Deakin University (allplay.org.au). AllPlay has the broad aim of creating new pathways for inclusion for children with disabilities to participate in activities in the community. The pilot projects discussed sit within the AllPlay Dance arm of the broader AllPlay program. Through the two projects, we aimed to develop inclusive strategies to better support the participation in dance for children with two specific disabilities – cerebral palsy (CP; 2018 project) and autism (2019 project). We employed a methodology which draws on a dance studies approach whereby the practices of dance, that is the teaching, creating, and analysis of dance material and dances are the site of the research. We describe the development of the dance class material and methods, the diffusion of authorship, and, in particular, the use of “buddies”; older and more experienced dancers, as methods of inclusion. We also consider the social elements of dance classes through describing unexpected situations that arose and observations we made, as a way of making sense of what took place in the classes, to inform our future projects, and perhaps the projects of other groups.

The work of AllPlay Dance is undertaken through collaboration between dance and psychology researchers. The research is also undertaken in partnership with professional ballet company, Queensland Ballet (Queesnland Ballet Company, n.d.; queenslandballet.com.au). This cross-disciplinary partnership has two broad aims: (1) to develop methods (a program) to enable children with disabilities to participate in dance activities and (2) to measure the benefits of community-based dance programs for children with disabilities. This paper is focused around the first of the two aims. The two aims are interlinked to work toward the long-term goal of allowing dance to be prescribe-able by healthcare professionals for children with disabilities. To enable the benefits to particular disability groups, we are developing and testing the methods and approaches to deliver those benefits. Central to the approach to the AllPlay Dance program is the idea that true inclusion in community-based programs has the potential to offer benefits to physical, cognitive, and psychosocial functioning as an alternative to or in adjunct with therapy in a clinical setting. Notably, the work of AllPlay Dance differs from dance therapy in that it aims to promote the inclusion of children with disabilities in community-based, recreational dance programs and provide equal access to their potential benefits, rather than develop disability-specific interventions aimed at addressing or fixing “problems.”

Around one in six children live with disabilities (Boyle et al., 2011), such as autism spectrum disorder, cerebral palsy, and intellectual disability (American Psychiatric Association, 2013). Mental health problems affect around 30–50% of this population, which is greater than the typically developing population. These mental health problems are known to be a major driver of poor quality of life for both the individuals with disabilities and their families (Johnson, 2009; Janssen and LeBlanc, 2010; Einfeld et al., 2011; Dahan-Oliel et al., 2012; Jonsson et al., 2017). Meaningful participation in community activities, such as sport and dance, is a means through which a reduction in mental health problems and an increase in quality of life may be achieved (Johnson, 2009; Janssen and LeBlanc, 2010; Dahan-Oliel et al., 2012). Despite this, evidence-based programs that effectively promote community participation and improve mental health and quality of life for the whole family unit continue to fall short (Howells et al., 2019). Children with disabilities often do not have the same opportunities as their typically developing peers to participate in sport and dance and often struggle to find inclusive programs to cater to their individual needs. AllPlay was developed to address this gap, and over the past 5years, the AllPlay team has explored the barriers and facilitators to community sport participation for children with disabilities (Sivaratnam et al., 2020).

Dance studies involve the articulation, in writing, of the recognition that a dance or dancing existed (Giersdorf and Wong, 2019); a taking into account of the fact that the act of dancing is experienced in the body. Also present in our reporting about the two AllPlay Dance projects is the emerging development of a working method, that places equal weight on the approaches by the two disciplines of Dance and Psychology. As mentioned above, the work is based on a collaborative approach to the delivery of the program and the measurement of the work. Although collaboration across disciplines in the academy is common, it has been the case in AllPlay Dance that careful work and negotiation has been needed when combining discipline-specific knowledge and practices. As suggested by Calvo-Marino (2010), in order to undertake successful science-art collaborations, it needs to be understood that particular disciplines often take a different approach to their research. It is also often the case that there are differing aims for the outcome of the research. In the case of the pilot projects we discuss, our dual focus was to carefully develop a collaborative and accessible dance program, while at the same time create an opportunity to measure whether such programs offer children with CP and ASD similar benefits to those that have been demonstrated in the wider dance literature (e.g., Quiroga Murica et al., 2010; Burkhardt and Brennan, 2012). Collectively, these areas of focus work in concert to deliver our overall aim of working toward dance being prescribe-able for children with disabilities. Through our collaborative work, we have come to understand that employing the open-ended approach from dance studies based research allows us to continue to adapt and develop our work with the participants. We employ the “less instrumental values of creative inquires” (Hansen, 2017, p. 33), which allows us to measure our programs while concurrently asking what we still have to learn and how we can improve our work. We have chosen to report in this journal using a dance studies approach as a way of deliberately highlighting what we see as both the complexity and the advantage of working in this way.

We will describe the approach to the development of the content for the classes in the two projects and then focus on elements of the projects through three themes. The themes were identified through referring to existing theory as per a dance studies approach (Giersdorf and Wong, 2019) in which emerging understandings can be made sense of or critically evaluated through the application of the current thinking in aligned fields. Our three themes are as follows:

1. The premise of dance as a social practice in which participants inter-subjectively generate meaning and sense making informed by the methods of De Jaegher (2013).
2. The participation of older and more experienced buddies with the aim of supporting the inclusion of the children with disability.
3. The facilitation of a diffusion of authorship in the making of group dances as a tool for inclusion whereby children not only contributed to the creation of movement material but to the implicit and explicit decision-making about the dance they would perform (Millard, 2013).

The three themes are interlinked, and the discussion is developed across the themes citing instances in the classes.

## Project Planning and Development

In developing and reporting on our work, we acknowledge the significant existing field of research in the area of dance and disability and that the work of performance companies, such as Candoco, Cleveland Wheel Company, Restless Dance Company, and many others inform our own, particularly in our aim to challenge the existing aesthetic framework through which dance performance is viewed (Cooper Albright, 1997, p. 77; Restless Dance Theatre, n.d.). As suggested by Cooper Albright and Brandsetter (2015, p. 4), it is not possible to discuss dance and disability without also entering into a discussion about the ideologies of dance, expectations of those who watch dance and how the language used to describe dance, despite the work mentioned above, still tends to “denounce” the disabled dancing body as somehow less than its abled peers. Our aim is to make available the benefits of dance for children with disabilities, and we have approached our work from the point of view that each dancer has agency in their own dance and dancing (See page 12). We hope that the enabling of agency also offers the participants (and their audience) a release from those disempowering notions of *how* a body should dance. As we will suggest, our projects have offered opportunities for the dancers with disabilities themselves to question our notions/perceptions of the dancing, watched body. Cooper Albright (1997, p. 57) asks whether “…the integration of disabled bodies into … dance result in a disruption of “ableist” preconceptions about professional dance? Or does the disabled body “transcend” its disability to become a dancer?” Although the dancers with disabilities in our pilot projects were young and not professional, we approached our work from the point of view that transcendence is not only possible, but that it is assured because we consider each participant a dancer in their own right. This consideration is also supported through our method of enabling agency, for each participant in the way they dance.

The dance classes for the two projects were developed and taught by author Olivia Millard and drew upon her 20-year experience teaching dance in tertiary institutions, as well as her work with dance companies for dancers with diverse abilities including the Delta Project (2012, 2014) and Weave Movement Theatre (2014; Millard, 2020). Millard (2013) involved the participation of a group of individuals in a long term, regular dance practice that culminated in an improvised performance. Millard asserts that the approaches to dance making employed, including a practice of dancing with and watching each other, resulted in a diffusion of authorship (2013). Millard’s work informed how we approached the activities undertaken (see details in Project and Workshop Structures) and in the adoption of the method of watching other group members dance, in order to deliberately or non-deliberately appropriate the movements observed.

For both pilot projects, we also consulted with experts and members of the dance and disability communities seeking input in the planning stages, during the classes themselves and following the final performances. For the CP project, we consulted with performer and dance maker Dr. Melinda Smith OAM, who lives with cerebral palsy. Smith generously reviewed our written plans, attended one of the classes and offered subsequent written feedback, and attended the final performance. For the autism project, we sought input from community dance teachers and studio owners, scholars who had worked in the area of dance and disability and parents of children with autism who had previously participated in dance programs. We also talked with parents and the buddies regularly throughout both projects as well as observing the children themselves and where possible aimed to accommodate individuals or improve the delivery more generally. As is the case with any creative process in dance, we approached our work in an open-ended way, aiming to respond to possibilities as they arose and making adjustments where they were required.

The first of the two projects was developed for children with cerebral palsy, and the second was for participants with autism spectrum disorder. Cerebral palsy refers to a group of disorders of movement and posture that arise from a disturbance to the immature brain; they cause activity limitation and are also often associated with broader difficulties in non-motor domains (Rosenbaum et al., 2007). Autism, on the other hand, is a neurodevelopmental disorder characterized by social and communicative impairments as well as stereotyped or delimited patterns of interests, activities, and behaviors (American Psychiatric Association, 2013). While the benefits of dance participation for children aged 7–12 with CP and autism were rigorously measured as part of these projects, these data are currently being prepared for publication elsewhere.

### Program for Children With Cerebral Palsy

In September and October 2018, we undertook a pilot project in which nine children with CP participated in a series of dance workshops with a group of 17 “elite” classically trained dancers who volunteered to act as the “buddies” for the children with CP. The buddies attended a Buddy Briefing Session, which included an overview of CP (e.g., subtypes, common causes, symptoms, and treatments), information about being an AllPlay buddy, for example, how to be a buddy, what to do in classes, examples of what to expect, and strategies to help maximize participation.

The workshop series included four 45-min classes over a two-week period and culminated in a public performance in which the children with CP performed, accompanied by their buddies. In addition to our focus on the development and assessment of the class material, we aimed to determine whether our newly developed program was feasible and acceptable to this community and to gather initial information about the potential benefits of participating in dance for children with disabilities. We were also interested in the social impact on their buddies. The project took place in the dance studios at Deakin University and was a collaboration with Queensland Ballet and its Artistic Director Li Cunxin (author of Mao’s Last Dancer, Cunxin, 2003). The performance was also a launch of the AllPlay program and included a classical ballet masterclass for the elite buddies, taught by Li Cunxin.

### Program for Children With Autism Spectrum Disorder

The project for children with autism ran from July to December 2019 and was a randomized wait-list control trial (RCT) with significant pre- and post-testing. The trial was registered with ANZCTR in May 2019. Participants were allocated to either an *intervention group* or a *wait-list control* group. Children in both groups engaged in an 8-week dance program in which they undertook one, one-hour class per week. In each of the classes, they were supported by older dancers/buddies. The 8-week program culminated in a final rehearsal session and performance for family members. The workshop structures built upon and expanded those developed for the CP program. The following information was provided to the buddies in their briefing session:

- Every child with autism spectrum disorder is different, there is no “one size fits all.” Children with autism typically have difficulties with socializing and communicating with others.
- Although children with autism may have social difficulties, they are usually very keen to join in, but they might not know how. Some kids with autism might have lots of language and others might only use a few words or no words at all.
- Children with autism may like things to be done in a particular way or order, they may have a favorite activity that they are happy to do over and over again, and they may find it difficult to switch between tasks quickly or without much warning.
- Some kids with autism may find loud noises or particular sounds or textures uncomfortable. As every child is different, it is important that the teacher gets to know each child’s likes and dislikes, to be able to make dance as inclusive as possible for all kids.

As with the CP program, we also provided information about what to expect as a buddy and strategies to help the children participate.

While both of these projects were structured in a way that enabled us to collect data on feasibility, acceptability, and preliminary benefits to participation, the purpose of the current paper was not to detail the scientific aspects of the study but to describe the development of the program, discuss considerations for inclusion, and explore dance as a social practice through our impressions as the facilitators of the program.

## Materials and Methods

### Project and Workshop Structures

The AllPlay Dance projects were developed as a series of dance classes in which participants worked with set or learned movement material, dance improvisation, and tasks for movement generation in order to collectively create a dance for performance. Rudolph Laban’s Movement Principles within the framework of *Body, Space, Energy and Time* were employed as a structure for dance teaching and choreography, in order to develop a transferable and scaleable program (Laban, 1950). Rudolf Laban was a dancer, choreographer and movement theoretician, and one of the early pioneers of European Modern Dance. Laban developed a method of movement or dance analysis and a system of dance notation. The movement analysis work of Laban is ubiquitous in dance education and dance creation; even if it is not always acknowledged, it is often utilized without its users knowing its origins. The language and systems of Laban that we chose to use as a starting point for the creative aspects of our workshop structures are familiar to many dance teachers and practitioners. Our reasons for using them were both because they are comprehensive and adaptable principles which support naming, analyzing, and generating movement, even 70years after their publication, and their ubiquity means that the program we developed using them would then be in terms that are familiar for most teachers of dance.

Alongside Laban’s principles, we used a practice of improvisation developed by one of the authors of this paper, Olivia Millard, over several years. Improvisation, as described by dance researcher Hermans (2018), can be seen as a way to explore movement and rhythmic possibilities, to experiment with quality and narrative, and to cooperate with fellow dancers. Moreover, it provides an environment to “play, to try and to experiment” (2018, p. 2) free from expectations of fulfilling movement skill or coordination requirements. As described by Benjamin (2002, p. 7), leading practitioner in dance and disability and past Artistic Director of dance company Candoco, “[t]he capacity of dance improvisation to accommodate different bodies and its freedom from preordained steps makes it highly accessible.” In short, there is no “wrong” way to dance when improvising.

Our improvisation practice uses what are named “scores” as a tool for the generation of movement material and experiences. Scores in this practice are verbal propositions that enable rather than dictate the movement that comes when using them. There is not necessarily a straightforward, causal relationship between a score, the way we use a score and the dancing we do when we practice with a score (Millard, 2015). Nevertheless, scores enable us to ask questions about what is possible while dancing and allow each dancer to generate own dance.

Dancers who are very experienced in executing known or “set” movements, such as some of the highly trained elite buddies in the CP program, may initially feel anxious about improvising because they do not have a set vocabulary to draw upon. They may also not be accustomed to moving “spontaneously.” Using scores and utilizing Laban’s principles as starting points for the score supported an entry into dancing for all members of the group. This contrasts against a focus on perfecting steps and sequences, which many of these children find difficult, in particular the physical execution of steps, the ability to efficiently learn and remember new material, and the ability to monitor traditional spacing while keeping pace with their peers. In contrast however, we know that things like structure and routine are incredibly helpful for supporting the difficulties with thinking and behavior that many of these children experience. Incorporating scores and having some sort of underlying structure and starting point would have been very helpful for these children, as would being able to brainstorm ideas and engage in creative tasks with support and guidance from their buddies. Without the use of scores and the Laban structures, many participants may have found the open-ended nature of improvising too overwhelming and stressful.

#### Class Structure

The class sections remained the same, and more or less in the same order, for the whole series of classes. We kept a “timetable” stuck up on the wall with an arrow pointing to the section of the class we were in. This was especially important in the autism program because some of the children in those groups liked to have a good understanding of what was happening. If we forgot to move the arrow when we moved on to a new stage of the class, there was always a participant willing to remind us to change it!

##### Warm-Up

There were two aspects to our warm-up: simple set movement material and improvisation. Both aspects had the multiple purposes of bringing the attention of the participants into their dancing bodies, warming the body, and foreshadowing movements and techniques that would be utilized later in the class. The warm-up was increased in length and complexity over the weeks.

##### Set Material

The set material was executed with a metronome or soundtrack with a strong pulse to keep a consistent tempo. The material began with movements such as walking back and forward and clicking or clapping, while gradually adding more of the body into the movement, such as curving the spine and moving in and out of the floor.

##### Improvisation

To start, we named and then tried out various “energies” in our dancing bodies (Laban, 1950). We then built on that through asking questions about body parts, speed, and direction in conjunction with dancing with a certain energy. The energies initially used were suspended, percussive, vibratory, collapsing, and exploding. Once dancing, options for variation and change become more available. Important to explain here is that it was essential to insert the teaching, dancing body in the situation – whereby the teacher was constantly demonstrating while also conveying information verbally. We began by introducing an energy and then very briefly discussing how that might play out in dancing before launching in to moving all together; trying out what that energy might mean in each of our dancing bodies. While dancing, the class leader would talk, sometimes describing what she was doing sometimes making suggestions or asking questions about what might be possible. The dancers were encouraged to move continuously. They did not need to stop dancing to listen to suggestions or to think of what they might do next, rather, they could add to or change their dancing in response to ideas that arose.

The following questions (adapted from Laban’s principles) were asked while improvising:

- Which part of the body moves?
- In which direction or where in the space does the movement take place?
- What is the speed of the movement?
- How much energy is used? What kind of energy is it?

Improvisation was not only used as a warm-up method. We also used improvisation while making small dances in groups, and it was included in the final performance, often as a way for dancers to travel from one part of the stage to another.

##### Learning Material

A series of short phrases were developed and taught over the weeks. With each new phrase or step, there were always options for the way it could be executed and time given to adjust the movement or create a new version of it.

##### Making Material

In groups, from about the third or fourth week, participants generated their own movement phrases using various tasks such as making new versions of existing sequences, using movement words to create new sequences, and using improvisation scores to structure dance sequences.

Each week participants also had a chance to revise, practice, and build on work from previous weeks. Where appropriate, movement material was videoed and shared to aid retention from week to week. Dancers were encouraged to keep the sequences with named movements the same in order to keep continuity across the whole group, but also so that we would have a mechanism (the original sequence) for remembering the material they had made.

It was always an option for the dancers to adapt movements to suit/enable all dancers to achieve a version of each word. We also encouraged the dancers to create completely new movements for the words if they desired. Predictably, there was a great range of interpretation of the instructions: Some groups kept their material much the same as the original and varied the timing or the pathways that they travelled on, and others used the original sequences as a kind of holding place and made smaller or larger changes here and there. Some groups made completely new phrases, keeping only the words as guides. As would be assumed, some groups, such as those who had dancers in wheelchairs or walkers, made complex and layered changes, which included dancing with props (scarves) and having different dancers doing different movement with varied timing.

##### Showing/Watching

Each week, there was a chance to show the material made in small groups while the rest of group watched. Observers were encouraged to watch and identify movements they recognized. Over the weeks, the material built in complexity and worked toward the final dance performance. Observing also aided group decision-making, specifically the shape the dance should take.

## Discussion

Although we undertook careful plans for the development of the two projects and consulted with experts as appropriate, there was much we could not anticipate about how individuals might engage with aspects of the program. Not all children responded in the same way to each section of the classes. For example, in one autism session when most of the members of the group appeared to be thoroughly engaged in improvising to music, one child called out “[t]his is boring.” We acknowledged the “boring” and messy moments, we changed approaches for children who seemed unable to join in, and we made space for children who needed to rest. This discussion takes into account the planned elements of our project, including the use of buddies and the diffusion of authorship to support participants to feel included. It also describes unexpected events and observations; aspects of the projects that changed what we understand, which will inform our future planning. As stated above, the three themes were developed through the application of existing theory, although they were equally informed by what took place in the present of the classes.

### Intersubjectivity and the Social Practice of Dancing

Socially engaged art practice, or social practice in art, refers to work in any artform in which there is a communal or collaborative approach to undertaking an artistic practice or the production of work. Work in this area is often engaged in activism or embedded in particular community projects. Social practice in art arose from a desire to shift from the finite creation of a closed “object,” particularly in visual art. There are many examples of this kind of work, as described by Claire Bishop (2012, p. 77): the outcome-free *dèrive* (drifting) of artists involved with *Situationist International* in the 1950s and 60s (2012, p. 77) and the *Sonsbeek* projects (2012, p. 205) that were named as activities rather than exhibitions (Millard, 2017). Other examples include work such as the “situations” constructed by Tino Seghal in which the relationships between art and spectator are challenged. As described by art critic Nicolas Bourriaud, these works involve an “intersubjective exchange” (Jackson, 2011). In listing this work, we do not suggest that the AllPlay Dance projects necessarily lie in this canon. Nevertheless, the parallel is useful because our work includes intersubjective sense making, has a dance or artistic practice as its primary goal (rather than therapy itself), and is also only possible because of the social interactions upon which it is founded.

Philosopher of mind and cognitive science, Hanne De Jaegher, has an interest in the relationship between how we understand each other, our interactions and how we see and understand the world. Intersubjectivity, defined by De Jaegher (2016, p. 393) as “participation in the investigation of how experience transforms when examining it together,” enables the development of perspectives that are affected by more than one participant. The experiences exist in our “embodied habits, attitudes and comportments” and are co-created (De Jaegher et al., 2016, p. 492). De Jaegher describes particular conditions in which intersubjectivity is “graspable.” She writes that those kinds of interactions involve “…two or more autonomous agents co-regulating their coupling with the effect that their autonomy is not destroyed and their relational dynamics acquire an autonomy of their own.” Examples De Jaegher use are conversations, collaborative work, arguments, collective action, and dancing (2017, p. 491).

Social interactions are multilayered. They involve communication that is verbal and non-verbal and direct or indirect demands in complex and changing time scales (De Jaegher et al., 2010, p. 442). Through completing a variety of tasks, participants in the AllPlay Dance projects were involved in social interactions that had dancing and dance making as their primary purpose. Although there was time to “chat” briefly at the beginning of each session, we spent most of the session engaged in the dance activities. As described by De Jaeghar, the participant’s experience exists and is shared in the body and embodied actions, as part of an exchange, such as improvising together, as well as the more obvious interactions such as negotiating to create movement material. Examples of interactions that were undertaken as part of dancing are as follows: watching each other to synchronize the timing of a movement, “feeling” another’s rhythm to dance together, appropriating the movement of others through watching and dancing, verbal interactions in making material and sharing ideas, and touching each other as part of movements and lifts. The intersubjective sense making unintentionally undertaken by the participants allowed them to assert themselves as individuals in the situation particularly because the primary focus of that situation was something other than social interaction, that is, it was to dance and to collectively create dances.

#### Creating Dance Sequences

As described above, both of the projects (CP and autism) included workshop situations in which the participants worked together to create movement material, either improvised or set, and small group dances to be included in the larger dance. Undertaking a dance making task is complex, particularly in a group, because it involves following a particular set of instructions, responding creatively to those instructions, finding a physical solution to the task which needs to be learned and remembered, and then being watched while performing the results of that task. In our workshops, many of the group dances that were created included multiple compositional elements such as different material being danced at once, variations in timing, lifts or partner work and travelling in multiple directions. In order to create, remember, and perform those dances, each member of the group needed to engage in complex physical and verbal social interactions, to initiate, and to accept suggestions. The main focus of the creative task meant that any social negotiation happened within the framework of the dance creation.

Dance researcher Sherrie Barr (2015, p. 58) writes that a dance which is made collaboratively is a direct result of the interactions that take place, through a “give and take” in the creative process. Not only were the dancers in our projects engaged in creating dances in groups, they were creating dances that were only possible to create in that particular situation and with those particular individuals. Barr writes that “… the emergent dance is not separate from its individual community members but rather created in concert between them and the concerns of the specific community” (2015, p. 58). Placing the dance creation itself at the center of an interaction also enabled social choices and interactions that may have otherwise been difficult for some individuals to participate in. It may also be possible to retrospectively reflect on the social interactions and to develop perspective or an understanding of them, through the lens of the dance making itself.

#### A Discussion About Costumes in Four Parts

When we were running the second of the two autism groups, the dancers participated in a discussion over a period of 4weeks about what their costumes would be. In working with the previous group, it had become evident that, for some children, performing in a costume was very much part of their expectations of participating in a dance performance. While we certainly did not have the time (or the desire) to produce costumes, and it was not part of our approach to dictate what the dancers should wear, we entered into a discussion about the costumes with 5weeks to go. The discussions were not planned but rather unfolded as a forum in which all members of the group could share their suggestions and desires. While it did not matter at all what the dancers wore in the end, it became very important to take the suggestions seriously and to work toward an agreed cohesion, while still enabling every individual to dress in the way they wanted to. We arrived at a framework which included the following: woodland theme with a particular color scheme; Christmas; Harry Potter; magic; colored socks. In retrospect, it seems very chaotic but the extensive discussion that underpinned the development of the theme included serious social interactions in which all members of the group took part, and every contribution was equally valued. Individuals could suggest and agree (or not) what costumes should be worn through discussion and interaction. De Jaegher (2013) describes social interaction as a “co-regulated coupling between at least two autonomous agents.” Without the situation being overtly social, intersubjective interactions were taking place in which individuals were participating in a series of negotiations through which their own interests were expressed while there was also an imperative to arrive at an agreement (Millard, 2020). Each member of the group, whether they were 7 or 22, arrived on the performance day ready with their carefully chosen costume, some more elaborate than others. Although the audience may have seen only what the dancers were wearing, the costumes symbolized the active and successful participation by all members of the group in a negotiation, with a mutually satisfactory result.

### Buddies

Crucial to the development and success of the program was the participation of “buddies” who were older and had an existing level of dance experience. The buddies ranged from 14 to 25 in age and had varying associations with dance such as vocational aspirations or plans to be teachers of dance. Far from merely being present to help dancers “learn” or adapt steps, buddies became mentors, creative collaborators, and friends to their dancing partners.

Kim Dunphy and Jenny Scott, authors of *Freedom to Move* (2003) which charts their extensive work with people with disability in the Dance Movement Therapy paradigm, recount different situations working with “assistants,” volunteers, and undertaking team teaching. An assistant, whose role is to support the leader of the workshop, can help keep the “magic” through minimizing distractions and is integral to running a successful program (2003, p. 193). The assistant can also provide “emotional support” as well as sharing in the joys of special moments (p. 195). With support from the leader, the assistant works toward leadership roles. Co-operating with professionals from other disciplines can offer opportunities for knowledge sharing, but it is important to be aware of a potential lack of alignment in intention, particularly in the real time situation of the class. Dunphy and Scott (2003, p. 203) suggest that volunteers can potentially be of great help, although it is crucial that they are consistent and committed. The name buddy for the older dancers who helped in the AllPlay Dance program implies that their role was to work with the participants themselves, rather than to assist the leader or teacher. It is a subtle distinction but crucial in articulating the work that they did. The buddies worked with one or two particular participants for the duration of the program, and as suggested above, their role was not to help their partners do “well,” but rather to support their inclusion, help them find their creative voice, and to help them enjoy the program.

Buddies recruited for the AllPlay dance projects met particular criteria. The buddies for the CP program, as mentioned above, participated in a Master Class with Artistic Director of Queensland Ballet, Li Cunxin. Queensland Ballet set the criteria for those buddies, which included usual participation in at least 7h of ballet training per week and an aspiration to become a professional classical dancer. The CP buddies had great skills as dancers, and many of them were also capable improvisors and experienced in composing dances. The emphasis of the autism program was not strictly on ballet, and the buddy population was much more diverse. The criteria for joining the program included aspirations to become a dancer or dance teacher and to be undertaking at least 7h of training each week. Buddies in the autism program included some of the CP buddies who wanted to be involved again, dancers from the undergraduate dance program at Deakin University, students working toward becoming dance teachers and some psychology and occupational therapy students with significant dance training.

In both programs, we aimed to keep the pairing consistent for all sessions. In some cases, this was not possible, particularly because some children missed a session or two. There was also some attrition of participants. In the CP project, the buddies outnumbered the participants with CP. Some dancers had three buddies, although others only had one. Although the difference in numbers was not planned, it turned out to be fortunate in the CP program because the dancers worked in small groups and created sequences with lifts and complicated configurations with those with wheelchairs or walkers.

The range of ages in the buddies meant that their approach to their work and their way of relating to their partners varied. Some buddies seemed to see their role as a teacher or mentor, some related to their partners as peers or friends. Their work involved encouraging confidence at times and at others working as collaborators in the making of dances. Upon arrival, some participants were shy, or reluctant to participate, others were incredibly excited. Some buddies practiced the dances over and over again with their partners, others listened, while their partners chatted about their ideas for the dances or about everyday happenings. Some participants wanted to hold their buddies’ hands, and others wanted help with handstands or other tricky moves. One participant performed an amazing knee slide that his buddy, though she tried, just could not emulate. All participants and buddies worked together to create and perform their dances.

For most of the children, for varying reasons, their buddy was their means to participation in the program. It is easy to imagine that a child with CP in a wheelchair has very different barriers to participation in a dance program than a child with autism. For that reason, there was no one “way,” in either of the projects, that a buddy should work, or interact with their partner. Instead they figured out, through social interactions over time, the best ways to work. Without exception, the buddies were generous and approachable, and the participants responded to that generosity.

Dancer Caroline Bowditch states that inequality for people with disability is a result of a lack of prioritizing of access and inclusion. She also states that responsibility for addressing the problem of inequality should not lie with people with disability alone, “We all have a responsibility and a role to play” (Bowditch, 2020, p. 53). We expect that the buddies we worked with will become the dance teachers and dance makers of the future. Having a generation of dance teachers who not only understand how to work inclusively with children with disability but who see it as a given in the way they approach their work may mean that there are more programs available for children of all abilities in the community, that taking responsibility for addressing inequality begins to become part of dance teaching practice, rather than an “extra” consideration (Bowditch, 2020). We may also have done some of the work of challenging the ableist preconceptions of how dance should look and what constitutes a well-choreographed dance.

### Diffusion of Authorship as a Method of Inclusion

One of our approaches to inclusivity was to enable all participants to have agency in the kind of movement they were doing. In almost every aspect of the class, the dancers could choose (to varying extents) what dance moves they did or at least how they did them. As described above, participants were also supported to generate their own movement material through naming and recreating movements with prompts based within the framework of Laban’s movement analysis (1950).

In the function of an author, according to Foucault (1994, p. 208), is the “plurality of the self.” The “I” of the author in a single work (or series of works) could include more than one function, such as the assertion of a particular will or opinion, the demonstration of an understanding or compliance to conventions, and display of a meaning or purpose. These “I”s can exist in the one work simultaneously where none of them is a real individual. In the case of the movement material and dances created in the AllPlay Dance workshops, the “I”s were many. Even though the workshops had a teacher or leader, and there was certainly a need to be organized and structured at all times, the underlying premise of a diffusion of authorship (that is the idea that each dancer could participate in the determination of the dancing they did) allowed multiple “I”s in the dances. These “I”s did not necessarily relate directly to individuals, but they resulted from individuals making choices about their dancing.

Sarah Whatley et al. (2015) examine authorship considerations, including in relation to copyright, of disabled dancer Caroline Bowditch in her short film *A Casting Exploration*. The film explores Bowditch’s experience of being re-cast in a role in dance work, *Love Games* by Cleville (2011). As well as discussing the role of “virtuosity” in creation and performance, dance and disability, Whatley et al. (2015, p. 72) ask “[w]hile the choreographer may compose the dance, why can the dancer not be considered as an arranger of that composition?” This discussion is important because Bowditch, through her “arrangement” of the choreography, through her dancing body, is both challenging the perception of virtuosity, and as suggested by Whatley contributing to the authorship, whether or not it is acknowledged (p. 72). An individual dancer, with individual experience, body and “ability” might be considered the author of their own dance or at the very least of their dancing. In the AllPlay Dance projects, our questioning of authorship, or rather our deliberate enabling of the diffusion of it, also aimed to challenge the perception of virtuosity. The performances created by the groups confronted the expectations of what a dancing body should do and how that might be perceived. What we were also doing was asking whether, through intentionally diffusing the authorship, through enabling the agency of each individual in the creation of the dances, we could provide an opportunity for children with disability to dance, that was inclusive.

The situations in which dancers had agency in their dancing varied in each class and throughout the projects. In improvising, dancers could choose whatever movement they wanted to do, although their dancing bodies were certainly structured by the environment in which they were dancing. In adjusting phrases that had been learned, the dancers could make versions or movements that suited their bodies and their desires. Some dancers added a slide to the floor whenever they could, others made versions of movement that were percussive or had jumping in them. When the small groups made set dances using words or scores, some groups used complex timing arrangements, some included lifts or balances, and some developed material with repetition or in which all members of the group danced the same movement. The buddies played a vital role in enabling each participant to make choices about their dance. They encouraged and supported their partners through making suggestions or helping them to try out ideas. As the participants became more confident, the role of some of the buddies was to help their partners realize their “visions” by stepping back or by becoming the executor of created movements.

As suggested by dance scholar Susanne Ravn, it is the dance situation itself which determines how one can have agency. Ravn (2020, p. 79) states that, “…agency is our capacity to engage in and form part of actions as these are aroused through the way we form part of situations”. In the AllPlay Dance projects, that meant that the participants were able to make their own dance movements, or versions of those movements, asserting their agency, because they were in a situation which foregrounded creative individual input to collaborative dance making. In this way, according to Ravn (2020, p. 79), situations such as the AllPlay Dance projects could be thought of as a “… kind of agentive training” in which participants are not only offered the opportunity to have agency but also practicing doing so.

### Collaborative Research

The two projects were only possible because of the collaborative approach to the research, that is, the coming together of two discipline-specific research practices and working methods. We employed the dance studies methodology of the taking into account of the occurrence of dance activities and their effects while concurrently gathering objective and subjective data from children and parents both prior to and following the dance program to measure its benefits. Performing Arts scholar Hansen (2017, p. 40) suggests that in order for science and creative arts researchers to successfully collaborate, they enter a “third space” that sits in between the methodologies of the two disciplines. The third space is a place for connection and discovery, which still allows the separate disciplines to generate outputs appropriate to their lines of enquiry (2017, p.40). In the projects that we have described, the third space emerged as the projects progressed. In seemingly simple situations such as supporting children who were disengaged or uncomfortable, members of the team worked together using methods that relied on existing experiences and knowledge: The dance researchers might try physical solutions such as demonstration or creative tasks, while the psychology researchers recognized social or behavioral indicators and responded accordingly. These strategies were often employed in concert with each other. Each of these situations was an opportunity for generating new understandings. Not only were we able to observe what was taking place through the intersubjective participation in dance and dance making activities, and measure the benefits of participation in dance, we are developing a shared language and approach to conducting dance activities for children with disabilities, which is not wholly of one or the other discipline but a combination of the two.

## Conclusion

As mentioned above, not all aspects of our projects ran smoothly. Some children left the projects after a couple of weeks. Others stayed although the classes were not exactly what they were hoping for. There were participants who had difficulty consistently participating, despite our and their parents’ best effort to find a “way in” for them. One parent was disappointed that their child had not been taught to dance “well” as they perceived it, instead in the performance they seemed to be dancing individually, and not in sync with the group. There was a suggestion that there was not enough social time, that too much of the time was spent dancing with little time left to make friends.

One short coming which we feel most keenly is that an eight-week program is too short. It is too short for there to be real development of physical and creative skills. Even more concerning is the fact that for several children, this was the first time they had been able to participate in a dance program, having been excluded from community programs because of a lack of confidence or training on the part of the teachers, or because the way the programs run are simply unsuitable. To offer a series of classes which children felt happy and confident in, and in which they had agency in their own participation, only for the classes to be over after such a short time, felt inadequate and disappointing for us; the real implications for the children are possibly akin to perpetuating their struggle to be included.

We continue our work, aiming to address the challenges and to continue to develop our program in varying ways. In 2021, we take on two new projects: one to develop and deliver a series of online dance classes for children with autism, the other to consult with community dance teachers to develop professional development so they are supported to include children with disability in their classes.

This paper discusses the development of two projects that preference the experiential understandings gained through a dance studies approach. Other than to reap the therapeutic benefits, is there value in encouraging children to dance, particularly if there is a chance of them not living up to an expectation of what a dancer is? While working to develop a program that is inclusive and transferable, could we also challenge the notion that dancers “overcome” their disability in order to “become” a dancer? Instead these young dancers could, in Cooper Albright’s words, “radically re-figure the very category of dancer itself” (1997, p. 65). By removing the authority of what dancing should be from a single individual and diffusing it among all participating members of a group, there is the possibility to define the dancer as one who considers themself to be dancing. Caroline Bowditch writes: “[w]e need to move away from seeing access as a drain on resources, time and quality toward seeing it as the most creative opportunity we have available” (2020, p. 54). Rather than aiming to “intervene,” in AllPlay Dance, we aim to continue working with children in a collaborative way, enabling them agency in their own dancing and therefore in their social and developmental goals.

## Data Availability Statement

The data sets presented in this article are not readily available because the data used in this article include the development of an original dance program for children with disabilities and qualitative responses to survey questions. Requests to access the datasets should be directed to OM, olivia@deakin.edu.au.

## Ethics Statement

The studies involving human participants were reviewed and approved by Deakin University Human Research Ethics Committee. Written informed consent to participate in this study was provided by the participants’ legal guardian/next of kin.

## Author Contributions

OM, EL, NR, and JM participated in the conception and design of the study. OM, EL, and NP were involved in recruitment and data collection. OM and EL conceived and designed the dance program and OM facilitated and taught workshops. OM analyzed the program, formulated the framework for paper discussion, and led the paper. All authors contributed to the article and approved the submitted version.

## Funding

AllPlay Dance is funded by National Disability Insurance Scheme, Moose Toys, MECCA Brands, Grace and Emilio Foundation, and the Wenig Family. Donations and philanthropic support from MECCA Brands, Grace and Emillio Foundation funded research staff and the Wenig Family funded PhD scholarship. NDIS supported initial AllPlay Dance program development including online resources (see allplaydance.org.au) and systematic review. Moose Toys funds the AllPlay program (allplay.org.au). The funders were not involved in the study design, collection, analysis, interpretation of data, the writing of this article or the decision to submit it for publication.

## Conflict of Interest

The authors declare that the research was conducted in the absence of any commercial or financial relationships that could be construed as a potential conflict of interest.

## Publisher’s Note

All claims expressed in this article are solely those of the authors and do not necessarily represent those of their affiliated organizations, or those of the publisher, the editors and the reviewers. Any product that may be evaluated in this article, or claim that may be made by its manufacturer, is not guaranteed or endorsed by the publisher.

## References

[ref2] American Psychiatric Association (2013). Diagnostic and Statistical Manual of Mental Disorders DSM-5. 5th *Edn*. Washington, DC: American Psychiatric Publishing.

[ref3] Barr S. (2015). Collaborative practice in dance research: unpacking the process. Res. Dance Educ. 16, 51–66.

[ref4] Benjamin A. (2002). Making an Entrance: Theory and Practice for Disabled and Non-Disabled Dancers. London and New York: Taylor and Francis Ltd.

[ref5] Bishop C. (2012). Artificial Hells: Participatory Art and the Politics of Spectatorship. London and New York: Verso.

[ref7] Bowditch C. (2020). “Access and inclusion,” in The Relationship Is the Project. eds. Lillie J. Larsen K. Kirkwood C. Jacki Brown J. (Australia: Brow Books)

[ref8] Boyle C. A. Boulet S. Schieve L. A. Cohen R. A. Blumberg S. J. Yeargin-Allsopp M. . (2011). Trends in the prevalence of developmental disabilities in US children, 1997-2008. Paediatrics 127, 1034–1042. doi: 10.1542/peds.2010-2989, PMID: 21606152

[ref9] Burkhardt J. Brennan C. (2012). The effects of recreational dance interventions on the health and well-being of children and young people: a systematic review. Arts Health 4, 146–161.

[ref10] Calvo-Marino B. (2010). “Neural mechanisms for seeing dance,” in The Neurocognition of Dance: Mind Movement and Motor Skills. eds. Bläsing B. Puttke-Voss M. Schack T. (Abingdon, Oxon; New York, NY: Taylor and Francis Group)

[ref12] Cooper Albright A. (1997). Choreographing Difference: The Body and Identity in Contemporary Dance. Middletown, CT: Wesleyan University Press.

[ref500] Cleville J. (2011). Love Games at the Pathways to the Profession Symposium’, 1 April 2012 Available at: https://joanclevilledance.com/portfolio/love-games/ (Accessed October 11, 2021).

[ref13] Cooper Albright A. Brandsetter G. (2015). The politics of a prefix. Choreogr. Pract. 6, 3–7. doi: 10.1386/chor.6.1.3_2

[ref14] Cunxin L. (2003). Mao’s Last Dancer. London: Penguin Books.

[ref15] Dahan-Oliel N. Shikako-Thomas K. Majnemer A. (2012). Quality of life and leisure participation in children with neurodevelopmental disabilities: a thematic analysis of the literature. Qual. Life Res. 21, 427–439. doi: 10.1007/s11136-011-0063-9, PMID: 22101860

[ref16] De Jaegher H. (2013). Embodiment and sense making in autism. Front. Integr. Neurosci. 7:15. doi: 10.3389/fnint.2013.00015, PMID: 23532205 PMC3607806

[ref17] De Jaegher H. (2016). Intersubjectivity in the study of experience. Constr. Foundations 11, 393–395.

[ref18] De Jaegher H. Di Paolo E. Gallagher S. (2010). Can social interaction constitute social cognition? Trends Cogn. Sci. 14, 441–447. doi: 10.1016/j.tics.2010.06.009, PMID: 20674467

[ref19] De Jaegher H. Pieper B. Clenin D. Fuchs T. (2016). Grasping intersubjectivity: an invitation to embody social interaction research. Phenomenol. Cogn. Sci. 16, 491–523.

[ref20] Dunphy K. Scott J. (2003). Freedom to Move: Movement and Dance for People with Intellectual Disabilities. Sydney, Philadelphia, London: Maclennan and Petty.

[ref22] Einfeld S. L. Ellis L. A. Emerson E. (2011). Comorbidity of intellectual disability and mental disorder in children and adolescents: a systematic review. J. Intellect. Develop. Disabil. 36, 137–143. doi: 10.1080/13668250.2011.572548, PMID: 21609299

[ref23] Foucault M. (1994). Aesthetics, Method and Epistemology: Essential Works of Foucault 1954–1984. ed. Rabinow. P. New York: The New Press.

[ref25] Giersdorf J. R. Wong Y. (2019). The Routledge Dance Studies Reader. London and New York: Routledge.

[ref26] Hansen P. (2017). “Research-based practice: facilitating transfer across artistic, scholarly and scientific inquiries,” in Performance as Research: Knowledge, Methods, Impact. eds. Arlander A. Barton B. Dreyer-Lude M. Spatz B. (London: Taylor and Francis Group), 32–49.

[ref28] Hermans C. (2018). Let’s dance: participatory sense making in an eight-year-old boy with autism. J. Dance Educ. 19, 23–33.

[ref32] Howells K. Sivaratnam C. May T. Lindor E. McGillivray J. Rinehart N. (2019). Efficacy of group-based organised physical activity participation for social outcomes in children with autism spectrum disorder: a systematic review and meta-analysis. J. Autism Dev. Disord. 49, 3290–3308. doi: 10.1007/s10803-019-04050-9, PMID: 31102193

[ref33] Jackson S. (2011). Social Works: Performing Art, Supporting Public. Routledge. Available at: https://play.google.com/store/books/details?id=JBXHBQAAQBAJ&rdid=book-JBXHBQAAQBAJ&rdot=1&source=gbs_atb&pcampaignid=books_booksearch_atb (Accessed May 8, 2020).

[ref34] Janssen I. LeBlanc A. G. (2010). Systematic review of the health benefits of physical activity and fitness in school-aged children and youth. Int. J. Behav. Nutr. Phys. Act. 7:40. doi: 10.1186/1479-5868-7-40, PMID: 20459784 PMC2885312

[ref35] Johnson C. C. (2009). The benefits of physical activity for youth with developmental disabilities: a systematic review. Am. J. Health Promot. 23, 157–167. doi: 10.4278/ajhp.070930103, PMID: 19149420

[ref36] Jonsson U. Alaie I. Löfgren Wilteus A. Zander E. Marschik P. B. Coghill D. . (2017). Annual research review: quality of life and childhood mental and behavioural disorders – a critical review of the research. J. Child Psychol. Psychiatry 58, 439–469. doi: 10.1111/jcpp.12645, PMID: 27709604

[ref37] Laban R. (1950). The Mastery of Movement. Plymouth: McDonald and Evans Ltd.

[ref39] Millard O. (2013). From score to work? Making a group, improvising a dance. PhD Exegesis Deakin University, Faculty of Arts and Education, School of Communication and Creative Arts.

[ref40] Millard O. (2015). “What’s the score? Using scores in dance improvisation,” in Brolga: An Australian Journal about Dance, 40.

[ref41] Millard O. (2017). “Dancing participation: observations of a long-term group dance improvisation practice,” in Brolga: An Australian Journal about Dance, 41.

[ref42] Millard O. (2020). Dance as a social practice: the shared physical and social environment of group dance improvisation. Idea J. 17, 335–349. doi: 10.37113/ij.v17i02.395

[ref43] Queesnland Ballet Company (n.d.). Available at: https://www.queenslandballet.com.au/ (Accessed March 18, 2021).

[ref44] Quiroga Murica M. Kreutz G. Clift S. Bongard S. (2010). Shall we dance? An exploration of the perceived benefits of dancing on well-being. Arts Health 2, 149–163. doi: 10.1080/17533010903488582

[ref45] Ravn S. (2020). Investigating dance improvisation: from spontaneity to agency. Dance Res. J. 52, 75–87. doi: 10.1017/S0149767720000182

[ref46] Restless Dance Theatre (n.d.). Available at: http://restlessdance.org/restless-dance-theatre/ (Accessed May 10, 2020).

[ref48] Rosenbaum P. Paneth N. Leviton A. Goldstein M. Bax M. Damiano D. . (2007). A report: the definition and classification of cerebral palsy April 2006. Dev. Med. Child Neurol. Suppl. 109, 8–14. PMID: 17370477

[ref52] Sivaratnam C. Howells K. Stefanac N. Reynolds K. Rinehart N. (2020). Parent and clinician perspectives on the participation of children with cerebral palsy in community-based football: a qualitative exploration in a regional setting. Int. J. Environ. Res. Public Health 17:1102. doi: 10.3390/ijerph17031102, PMID: 32050514 PMC7037465

[ref53] Whatley S. Waelde C. Brown A. Harmon S. (2015). Validation and virtuosity: perspectives on difference and authorship/control in dance. Choreogr. Pract. 6, 59–78. doi: 10.1386/chor.6.1.59_1

